# Hidden Diversity in the Populations of the Armored Catfish *Ancistrus* Kner, 1854 (Loricariidae, Hypostominae) from the Paraná River Basin Revealed by Molecular and Cytogenetic Data

**DOI:** 10.3389/fgene.2017.00185

**Published:** 2017-11-24

**Authors:** Ana C. Prizon, Daniel P. Bruschi, Luciana A. Borin-Carvalho, Andréa Cius, Ligia M. Barbosa, Henrique B. Ruiz, Claudio H. Zawadzki, Alberto S. Fenocchio, Ana L. de Brito Portela-Castro

**Affiliations:** ^1^Laboratório de Citogenética de Vertebrados, Departamento de Biotecnologia, Genética e Biologia Celular, Universidade Estadual de Maringá, Maringá, Brazil; ^2^Laboratório de Citogenética Animal e Mutagênese Ambioental, Departamento de Genética, Universidade Federal do Paraná, Curitiba, Brazil; ^3^Departamento de Biologia/Nupélia, Universidade Estadual de Maringá, Maringá, Brazil; ^4^Facultad de Ciencias Exactas, Químicas y Naturales, Universidad Nacional de Misiones, Posadas, Argentina

**Keywords:** *Ancistrus* genus, cytotaxonomy, species delimitation, candidate species, chromosomal evolution

## Abstract

Only one species of armored catfish, *Ancistrus cirrhosus* Valenciennes 1836, has been historically described in the basin of the Paraná River, from Misiones (Argentina). However, the ample variation found in the morphology and coloration of the populations sampled in the tributaries of the Brazilian state of Paraná makes it difficult to establish the real taxonomic status and evolutionary history of the *Ancistrus* specimens, suggesting that *A. cirrhosus* is not the only species found in this basin. By combining data on mitochondrial DNA (COI gene) and chromosomal markers from different *Ancistrus* populations, totaling 144 specimens, in the tributaries of the Paraná, and specimens from Misiones (type-locality of *A. cirrhosus*), we detected five distinct evolutionary lineages. All the specimens were 2n = 50, but had four distinct karyotype formulae. The results of the Generalized Mixed Yule Coalescent (GYMC) and the genetic distances (uncorrected *P*-values) between lineages ranged from 3 to 5%. Clusters of 18S rDNA were observed in a single chromosome pair in seven populations of *Ancistrus*, but at different positions, in some cases, in synteny with the 5S rDNA sites. Multiple 5S sites were observed in all populations. Overall, the cytogenetic data reinforce the genetic evidence of the diversification of lineages, and indicate the existence of candidate species in the study region. The evidence indicates that at least four candidate species of the *Ancistrus* may coexist in the Paraná basin besides *A. cirrhosus*. Overall, our results provide a comprehensive scenario for the genetic variation among *Ancistrus* populations and reinforce the conclusion that the true diversity of the freshwater fish of the Neotropical regions has been underestimated.

## Introduction

The inclusion of genetic data in studies of taxonomy and evolution has had a profound influence on our understanding of the unique diversity of species in the Neotropical region (Pereira et al., 2011, 2013). The combination of approaches has altered our perceptions of the region's biological diversity and contributed to an increase in the rate of discovery of new species, especially in cryptic lineages. In this context, the delimitation of species using DNA sequences can be highly efficient, enabling the more systematic documentation of species diversity (e.g., Yang and Rannala, 2010; Ence and Carstens, 2011; Fujisawa and Barraclough, 2013). For example, the Generalized Mixed Yule Coalescent (GMYC) method has been designed to delimit potential lineages using information on a single locus, that is, this method considers that the mutations arising in one species cannot spread readily into another species (Ahearn and Templeton, 1989; Barraclough et al., 2003; De Queiroz, 2007). The premise of the GMYC method is that independent evolution leads to the emergence of distinct genetic clusters, separated by longer internal branches, optimizing the set of nodes that defines the transition between inter- and intra-specific processes (Barraclough et al., 2003).

Fish are an excellent candidate group for the application of integrative taxonomical methods, which combine different lines of evidence (DNA sequences, chromosomal data, and morphological features) to define taxonomic status. Most surveys of Neotropical freshwater fish have focused on major river basins, even though a large proportion of the total diversity comprises small species, found in minor rivers and streams. These species are often highly endemic and occupy a wide variety of microhabitats, providing enormous potential for diversification (Viana et al., 2013). Castro (1999) referred to the identification of the cryptic diversity of small freshwater fish as a major challenge for Neotropical Ichthyology. The reliable definition of species and their ranges is also essential to conservation strategies (Angulo and Icochea, 2010). In this context, the rivers and streams of the Paraná River basin provide an interesting study area for the evaluation of the degree to which the taxonomic diversity of these environments has been underestimated.

The Ancistrini is composed of 29 genera, with a total of 217 recognized species (Fisch-Muller, 2003). With 69 species, *Ancistrus* Kner, 1854 is one of the most diverse Ancistrini groups, the second richest in species of the Loricariidae (Ferraris, 2007; Bifi et al., 2009; Froese and Pauly, 2017). Cytogenetic data on *Ancistrus* are still scarce, and restricted to species found in the basins of the Paraguay River in Mato Grosso, and the Amazon, in Manaus (de Oliveira et al., 2009; Mariotto et al., 2011, 2013; Favarato et al., 2016; Prizon et al., 2016). While the cytogenetics of *Ancistrus* species from other river basins are still unknown, considerable variability has been found in this genus, with diploid (2n) numbers of 34, 38, 40, 42, 44, 48, 50, and 54 chromosomes. Surveys of the upper Paraná River have revealed the presence of a single species of *Ancistrus* in this basin, identified as *Ancistrus cirrhosus* (Langeani et al., 2007). However, the considerable variation in the morphology and coloration observed in the specimens collected in the tributaries of the Paraná River hampers the reliable identification of the *Ancistrus* species found in this region. Thus, given the karyotypic diversity of *Ancistrus* and the assumption that cryptic diversity exists in the rivers and streams of the Upper Paraná River basin, we combine chromosomal data and DNA sequences to evaluate the taxonomic status of these populations.

## Materials and methods

### Biological samples

**Figure 1:**
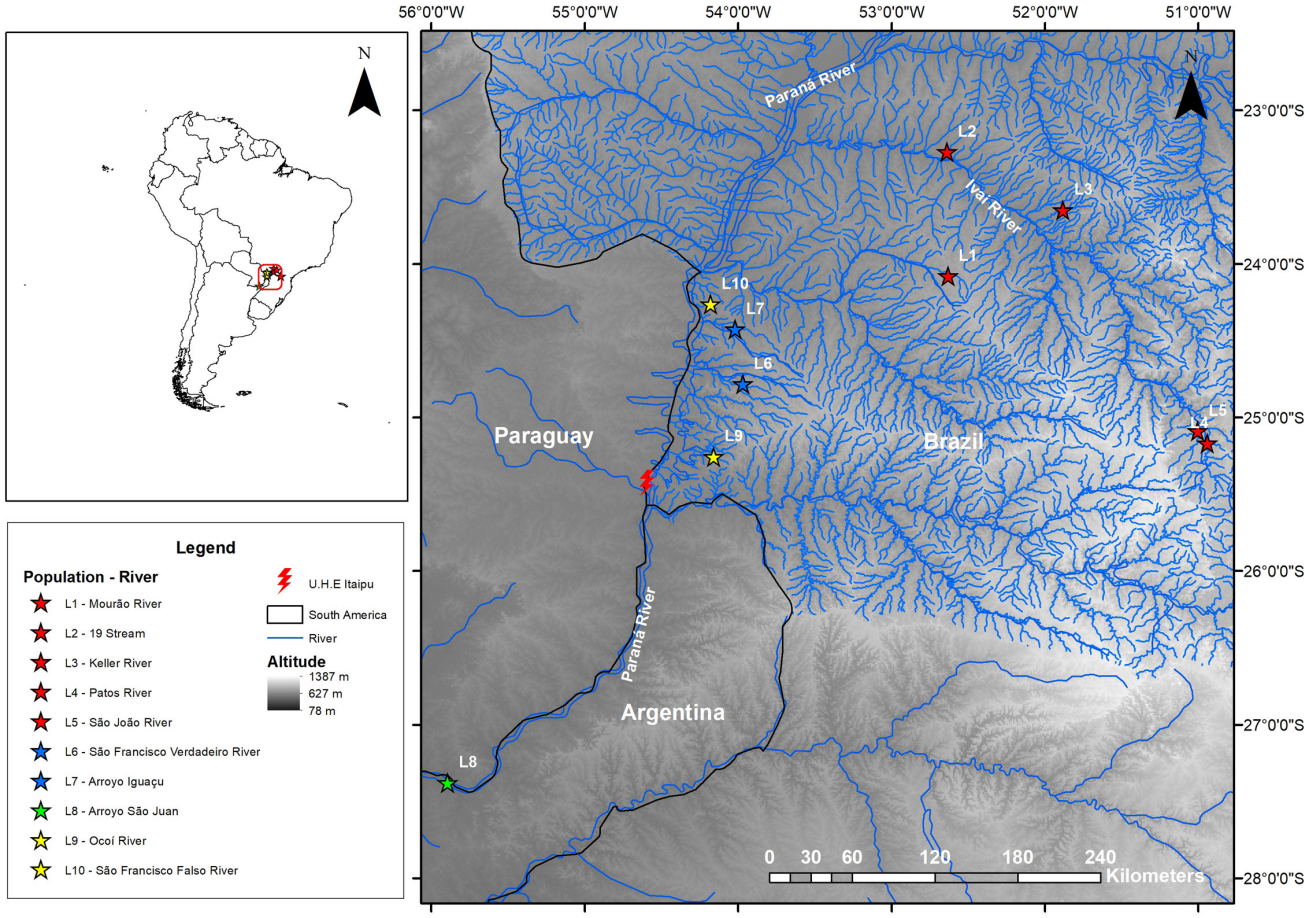
Topographic map showing the sites for the collection of specimens from the *Ancistrus* populations sampled in the Paraná River basin, in the present study. Each color represents a different clade recovered in our phylogenetic inferences (see Figure 2): red star, populations in clade I; blue star, populations in clade II; green star, populations in clade IV; and yellow star, populations in clade V. L1, Mourão River; L2, 19 Stream; L3, Keller River; L4, Patos River; L5, São João River; L6, São Francisco Verdadeiro River; L7, Arroyo Iguaçu; L8, *Ancistrus cirrhosus*; L9, Ocoí River; L10, São Francisco Falso River.

A total of 144 *Ancistrus* specimens were collected in ten rivers of the Paraná River basin (Table 1, Figure 1). Specimen collection was authorized by the Brazilian Environment Ministry through its Biodiversity Information and Authorization System (SISBIO), under license number 36575-1. The protocols used in this study were submitted to the Ethics Committee on the use of animals in research (CEUA) of the Universidade Estadual de Maringá (UEM) and approved under case number 013/2009. Voucher specimens were deposited in the ichthyological collection of the Limnology, Ichthyology and Aquaculture Research Center (Nupélia) at Universidade Estadual de Maringá, Paraná, Brazil. The catalog numbers are provided in Table 1.

**Table 1:** Details of the *Ancistrus* populations and specimens sampled in the study area of the upper Paraná basin.

**Code**	**Molecular sample**	**Cytogenetic sample**	**River**	**Locality/State or Province/Country**	**Geographical coordinates**	**NUP**
L1	1♂+1♀	13♂+36♀	Mourão	Campo Mourão/Paraná/Brazil	25°04′46″S, 53°54′45″W	11,993
L2	2♂	7♂+4♀	19 Stream	Paraiso do Norte/Paraná/Brazil	52°38′17″S, 23°16′08″W	13,646
L3	2♂+2♀	2♂+2♀	Keller	Marialva/Paraná/Brazil	23°38′48″S, 51°52′51″W	18,794
L4	1	–	Patos	Prudentópolis/Paraná/Brazil	25°09′59″S, 50°56′29″W	15,537
L5	1	–	São João	Prudentópolis/ Paraná/Brazil	25°05′10″S, 51°00′11″W	15,812
L6	1♂+1 Immature	2♂+2 Immature	São Francisco Verdadeiro	Toledo/Paraná/Brazil	24°46′50″S, 53°43′00″W	15,146
L7	1	–	Arroyo Iguaçu	Marechal Cândido Rondon/ Paraná/Brazil	24°25′18″S, 54°01′09″W	15,250
L8	4	2♂+10♀	Arroyo San Juan	Misiones/Posadas/Argentina	27°22.623′S, 55^o^53.571′W	18,795
L9	1♂+1 Immature	10♀+8♂+3 Immature	Ocoí	Medianeira/Paraná/Brazil	25°15′12″S, 54°01′55″W	4,729
L10	1♂+1♀	10♂+30♀	São Francisco Falso	Vera Cruz do Oeste/Paraná/Brazil	25°04′46″S, 53°54′45″W	15,145

*NUP, catalog number of the voucher specimen in the Nupélia collection; ♂, male; ♀, female*.

### Isolation, amplification, and sequencing of the DNA

Genomic DNA was extracted from the liver, muscle tissue, or a cell suspension of a subset of the sample (Table 1) using the TNES method, as applied by Bruschi et al. (2012). A fragment of the mitochondrial cytochrome C oxidase subunit I (COI) gene was amplified by polymerase chain reaction (PCR) using the primers: FishF1 (5′-TCAACCAACCACAAAGACATTGGCAC-3′), FishR1 (5′-TAGACTTCTGGGTGGCCAAAGAATCA-3′) (Ward et al., 2005). The solution for the amplification reaction included 20 ng/μl of the DNA template, 7 pmol of the forward and reverse primers, 10 mM of dNTPs, 1 U *Taq* DNA Polymerase, 1.5 mM MgCl_2_, and 1x PCR buffer (200 mM Tris, pH 8.4, 500 mM KCL). The amplification protocol was 5 min−94°C/(94°C/30 s−60°C/1 min−72°C/30 s) 35 cycles/10 min−72°C. The amplified products were purified using Exonuclease I (10 units) and SAP (1 unit), incubated for 45 min 37°C, followed by denaturation at 85°C for 10 min (Applied Biosystems, Santa Clara, CA, USA), as recommended by the manufacturer. The samples were then used directly as templates for sequencing in an automatic ABI/Prism DNA sequencer (Applied Biosystems, Foster City, CA, USA) with the BigDye Terminator kit (Applied Biosystems, Foster City, CA, USA), as recommended by the manufacturer. The DNA samples were sequenced bidirectionally and were edited in Bioedit version 7.2.5 (http://www.mbio.ncsu.edu/bioedit/page2.html) (Hall, 1999).

### Phylogenetic inferences and the delimitation of species

The phylogenetic relationships among the populations were inferred from the matrix of the 459-bp sequence of the COI gene. The dataset was complemented with 35 sequences of *Ancistrus* and one sequence of the sister group *Lasiancistrus* available in GenBank (Supplementary File 1). The outgroup was *Pseudolithoxus* sp., which was chosen based on the arrangement reported by Lujan et al. (2015). The sequence was aligned using Clustal W in BioEdit, version 7.2.5.0 (Thompson et al., 1994). The initial alignments were checked visually and adjusted wherever necessary. The dataset was used for phylogenetic reconstruction by Bayesian inference (BI) and the Maximum Parsimony (MP) approach.

Bayesian Inference (BI) method was applied to the dataset, which were divided into three partitions according to codon position for *mit*-COI. The best model of nucleotide evolution for each nucleotide partition was determined using Akaike Information Criterion (AIC) with the software jModelTest v2.1.6 (Guindon and Gascuel, 2003; Darriba et al., 2012). The BI was performed with the software Mr. Bayes 3.2.6 (Ronquist and Huelsenbeck, 2003), as available in the CIPRES Science Gateway 3.1 (Miller et al., 2010). BI was implemented using two independent runs, each starting from random trees, with four simultaneous independent chains, and performed 10,000,000 generations, keeping one tree every 1,000th generation. Of all trees sampled, 20% were discarded as burn-in and checked by the convergence criterion (frequencies of average standard deviation of split < 0.01) with Tracer v.1.6 (Rambaut et al., 2014), while the remaining were used to reconstruct a 50% majority-rule consensus tree and to estimate Bayesian posterior probabilities (BPP) of the branches. A node was considered to be strongly supported if it had a BPP ≥ 0.95, while moderate support was considered when BPP ≥ 0.9.

The MP analysis was implemented in TNT v1.1 (Goloboff et al., 2003) using a heuristic search method with tree bisection-reconnection (TBR) swapping and 100 random additional replicates. The bootstrap values of the branches inferred in this analysis were calculated with 1,000 non-parametric pseudoreplicates.

The genetic distances among and within species were calculated using the Kimura-2-Parameter (K2P) and *p-*distance model (Kimura, 1980) implemented in MEGA v 6.0. A neighbor-joining (NJ) tree of K2P distances was created to provide a graphic representation of the patterning of divergence between species with the software MEGA v 7.0 (Kumar et al., 2016). We also applied the General Mixed Yule-coalescent (GMYC) method to delineate species using single-locus sequence data. The GMYC requires a fully resolved and ultrametric tree as input for the analysis and combines a coalescence model of intraspecific branching with a Yule model for interspecific branching to estimate species boundaries and provide statistical confidence intervals to evaluate the sequences of the clusters recovered. Ultrametric trees were constructed by a BI tree in BEAST2 2.4.0 (Drummond et al., 2006; Drummond and Rambaut, 2007). We conducted three independent runs using different priors, that is, the Yule, relaxed clock, and constant coalescent models. An ultrametric gene tree was obtained for each prior. An XML file was produced using the BEAUti2 v2.4.5 interface with the following settings: GTR+G+I substitution model, previously inferred by MrMODELTEST (Nylander et al., 2004), empirical base frequencies, four gamma categories, all codon positions partitioned with unlinked base frequencies and substitution rates. The MCMC chain was 10 million generations long, and was logged every 1,000 generations. The Estimated Sample Sizes (ESS) and trace files of the runs were evaluated in Tracer v1.6. The resulting logs were analyzed in TREEANNOTATOR 2.4.4, with 25% burn-in, maximum clade credibility trees with a 0.5 posterior probability limit, and node heights of the target tree. The splits package (https://r-forge.r-project.org/R/?group_id=333) in R was used for the GMYC calculations, using the single-threshold strategy and default scaling parameters.

### Cytogenetic analysis

All specimens were anesthetized and euthanized by an overdose of clove oil (Griffiths, 2000). Mitotic chromosomes were obtained from kidney cells according to Bertollo et al. (1978). The AgNORs were revealed by the silver nitrate impregnation technique (Howell and Black, 1980). The regions of heterochromatin were determined by the C-banding technique (Sumner, 1972) and stained with propidium iodide according to the method of Lui et al. (2012). Physical mapping of the 5S rDNA and 18S rDNA sequences was carried out by fluorescence *in situ* hybridization (FISH) according to Pinkel et al. (1986), with probes obtained from *Leporinus elongatus* Valenciennes, 1850 (Martins and Galetti, 1999) and *Prochilodus argenteus* Spix et Agassiz, 1829 (Hatanaka and Galetti, 2004). We also isolated and cloned the rDNA 5S gene of the *Ancistrus* sample from Keller River using a more specific DNA probe for this gene. The genomic DNA extracted for the molecular analyses was used as the template for the amplification reaction, using the primers 5S-A (5′-TACGCCCGATCTCGTCCGATC-3′) and 5S-B (5′-CAGGCTGGTATGGCCGTAAGC-3′) (Pendas et al., 1994). The products of this amplification were isolated in the 1.5% agarose gel, then purified with an EasyPure® Quick Gel Extraction kit. These sequences were inserted into a linearized cloning vector (pJET1.2/blunt) by the CloneJET PCR Cloning kit (Thermo Scientific) and cloned in *Escherichia coli*. Ten clones with the insert containing the 5S rDNA sequence were selected for sequencing. The 5S rDNA nucleotide sequences were edited using BioEdit software and compared with sequences from GenBank database (www.ncbi.nlm.nih.gov).

Hybridization was conducted under high stringency conditions (77%), and the probes were labeled by nick translation with digoxigenin-11-dUTP (5S rDNA) and biotin-16-dUTP (18S rDNA). The 5S rDNA probes obtained from the recombinant plasmids were labeled by PCR using the fluorochrome digoxigenin-11-dUTP. The solution for labeling reaction included 20 ng/μl of the DNA template, 7 pmol of the forward and reverse primers, 4 mM of dNTPs, 1 mM of dig-11-dUTP, 1 U *Taq* DNA Polymerase, 1.5 mM MgCl_2_, and 1x PCR buffer (200 mM Tris, pH 8.4, 500 mM KCL). The hybridization signals were detected using anti-digoxigenin-rhodamine for the 5S rDNA probe and avidin-FITC (fluorescein isothiocyanate) for the 18S rDNA probe. The chromosomes were counterstained with DAPI. Double staining was carried out with chromomycin A3 (CMA3) and DAPI, according to Schweizer (1976). The metaphases were photographed using an epifluorescence microscope and adjusted for best contrast and brightness using the Adobe Photoshop CS6 software.

## Results

### Phylogenetic inferences and delimitation of species

**Figure 2:**
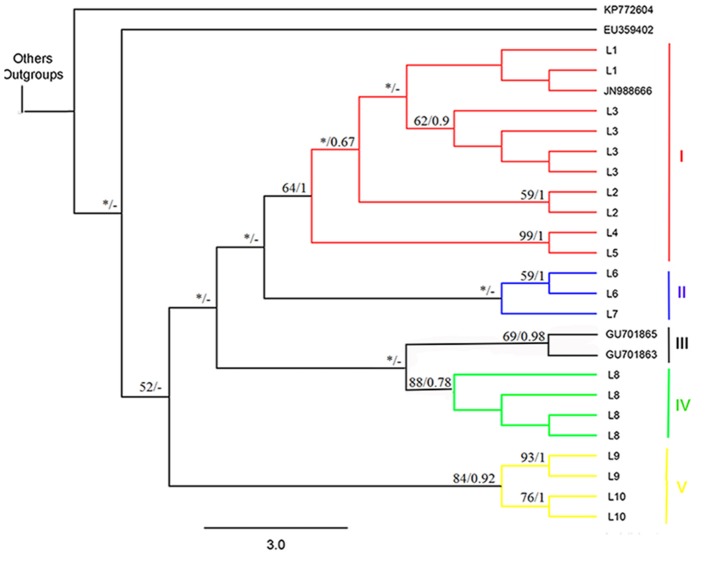
Strict consensus cladogram produced by the Maximum Parsimony analysis showing the intrageneric relationships of the *Ancistrus* species from the Paraná basin, based on a 459-bp sequence of the COI gene. The numbers above the branches show the bootstrap support and Bayesian posterior probabilities, respectively. Each color represents one of the five evolutionary lineages recovered in the analysis. The terminal species with alphanumerical identifiers were obtained from GenBank (Supplementary File 1). The asterisks indicate nodes with bootstrap values of < 50. The trace indicates that the node has not been recovered by Bayesian inference. Codes: L1, Mourão River; L2, 19 Stream; L3, Keller River; L4, Patos River; L5, São João River; L6, São Francisco Verdadeiro River; L7, Arroyo Iguaçu; L8, *Ancistrus cirrhosus*; L9, Ocoí River; L10, São Francisco Falso River.

**Figure 3:**
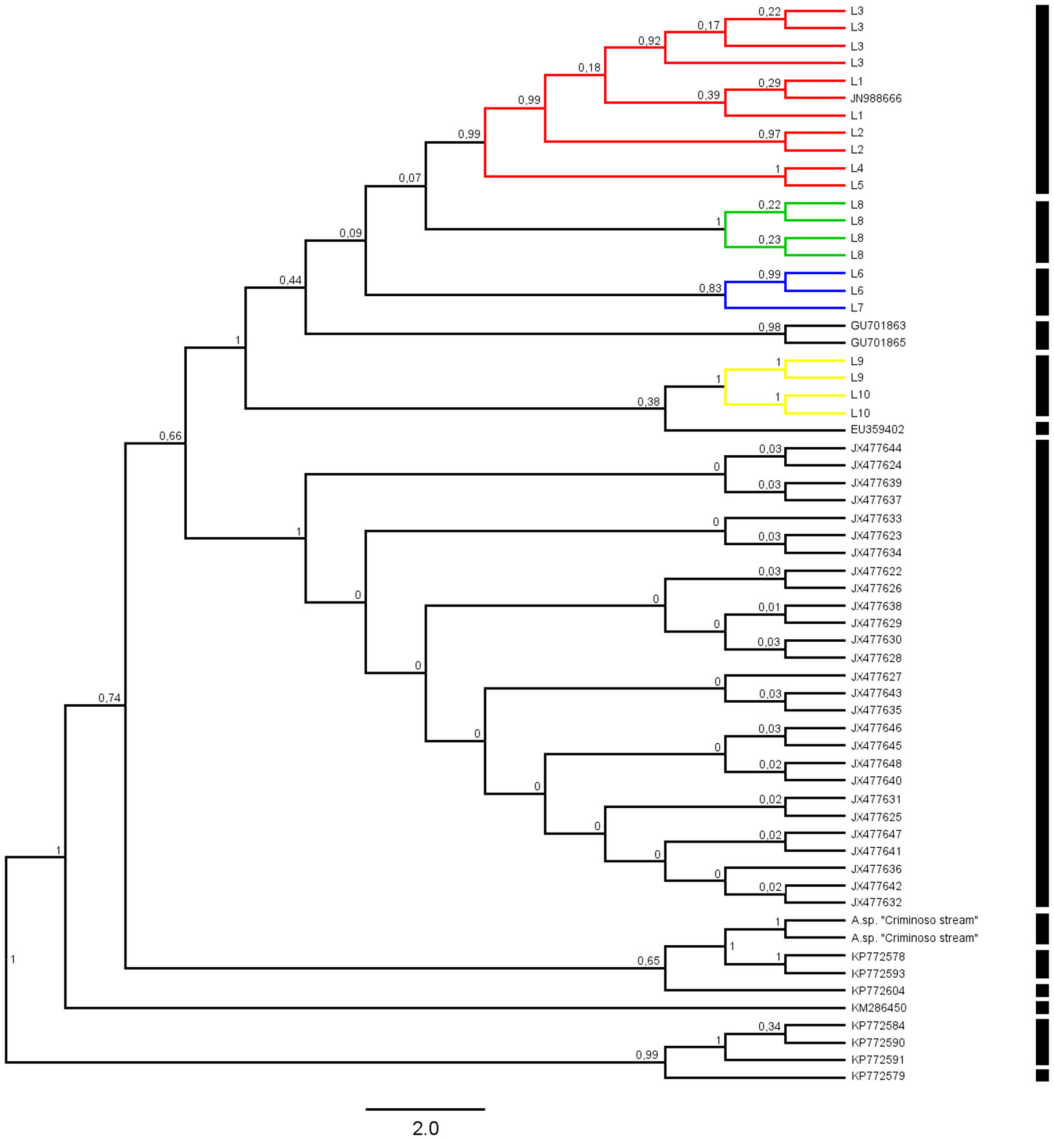
Bayesian consensus tree for the *Ancistrus* species of the Paraná River basin produced in BEAST2 2.4.0 and obtained from 10 million generations. Posterior probabilities are shown above the branches. Each color represents one of the five evolutionary lineages recovered. The results of the GMYC method using the Yule model of species delimitation are shown as black bars to the right of the phylogeny. Species access numbers are specified in the supplementary material (Supplementary File 1). Codes: L1, Mourão River; L2, 19 Stream; L3, Keller River; L4, Patos River; L5, São João River; L6, São Francisco Verdadeiro River; L7, Arroyo Iguaçu; L8, *Ancistrus cirrhosus*; L9, Ocoí River; L10, São Francisco Falso River.

The phylogenetic reconstructions based on the MP and BI approaches produced the basic topology of the dataset (Figures 2, 3). Topology inferred from the Bayesian analysis performed with the software Mr. Bayes 3.2.6. and from Neighbor-Joining were presented in the supplementary material (Figures S1, S2, respectively). These analyses recovered five clades from the *Ancistrus* populations of the Paraná basin. The first clade (clade I) comprises five populations: *Ancistrus* sp. “Mourão River” (L1) + *Ancistrus* sp. “19 Stream” (L2) + *Ancistrus* sp. “Keller River” (L3) + *Ancistrus* sp. “Patos River” (L4) + *Ancistrus* sp. “João River” (L5). The genetic distance analysis returned low uncorrected *P*-distances among these populations, ranging from 0.0 to 1.8% (Table 2). The second clade (clade II) included the *Ancistrus* populations from São Francisco Verdadeiro River (L6) and *Ancistrus* sp. from Arroyo Iguaçu (L7), separated by a genetic distance of 2.0% (Table 2). Two sequences deposited in Genbank as *A*. “*cirrhosus*” included in our dataset were recovered in the clade III. Clade IV included only the population from Arroyo San Juan (L8), Argentina, and was considered to represent the nominal *A. cirrhosus* due to its proximity to the type-locality of this species. Clade V consisted of the *Ancistrus* sp. “Ocoí River” (L9) + *Ancistrus* sp. “São Francisco Falso River” (L10) populations. The uncorrected *P-*distance between these populations (1.2%) was also relatively low (Table 2). In all cases, by contrast, the genetic distances (uncorrected *P-*values) between clades were at least 3%, being 3% between clades I–II, I–III, II–III, II–IV, III–IV; 4% between clades I–IV, II–V, III–V, IV–V and finally a genetic distance of 5% between clades I–V.

**Table 2:** Uncorrected pairwise distances between the mitochondrial COI sequences of the *Ancistrus* populations from the Paraná basin.

Populations^*^	**1 (%)**	**2 (%)**	**3 (%)**	**4 (%)**	**5 (%)**	**6 (%)**	**7 (%)**	**8 (%)**	**9 (%)**	**10 (%)**	**11 (%)**	**12 (%)**
1. *Ancistrus* sp. L1												
2. *A.cirrhosus* JN988666	0.5											
3. *Ancistrus* sp. L2	0.5	0.4										
4. *Ancistrus* sp. L3	0.5	0.4	0.4									
5. *Ancistrus* sp. L4	1.6	1.8	1.8	1.8								
6. *Ancistrus* sp. L5	1.6	1.8	1.8	1.8	0.0							
7. *Ancistrus* sp. L6	1.8	2.0	1.8	2.0	2.7	2.7						
8. *Ancistrus* sp. L7	2.2	2.5	2.5	2.5	3.3	3.3	2.0					
9. *Ancistrus cirrhosus* L8	2.5	2.7	2.7	2.7	2.7	2.7	2.2	3.1				
10. *A.cirrhosus* GU701865	2.2	2.4	2.4	2.4	2.7	2.7	1.8	2.7	2.2			
11. *A.cirrhosus* GU701863	2.0	2.2	2.2	2.2	2.9	2.9	2.0	2.9	2.4	1.3		
12. *Ancistrus* sp. L9	3.2	3.4	3.4	3.0	3.4	3.4	2.8	3.7	3.2	2.6	2.8	
13. *Ancistrus* sp. L10	2.7	2.9	2.9	2.9	3.1	3.1	2.4	3.3	2.5	2.4	2.5	1.2

* *Codes: L1, Mourão River; L2, 19 Stream; L3, Keller River; L4, Patos River; L5, São João River; L6, São Francisco Verdadeiro River; L7, Arroyo Iguaçu; L8, Ancistrus cirrhosus; L9, Ocoí River; L10, São Francisco Falso River. JN988666, GU701865, and GU701865: GenBank Access Number*.

The ultrametric trees obtained using the Yule and Constant Coalescent priors were congruent in the GMYC analysis, recognizing 13 separate entities and identifying nine clusters in our dataset (including outgroup sequences). Considering only the *Ancistrus* populations, the focus of the present study, the sequences were grouped into five well-supported clusters (Figure 3), which corresponded exactly to the major clades recovered in our phylogenetic inferences (BI and MP). The Yule and Constant Coalescent priors of the branching rates indicated that the likelihood of the null model (i.e., that all the sequences belong to the same species) was 412.5381 and 421.5504, respectively, with a likelihood of 421.9409 and 430.6968, respectively, for the GMYC model (i.e., the existence of distinct species). The difference is highly significant, indicating the presence of more than one species in our sample. The analysis based on the Relaxed clock priors presented low scores in Tracer and did not produce a reliable interpretation of the phylogenetic relationships among the populations, and was not considered in the species delimitation tests.

### Cytogenetic analysis

**Figure 4:**
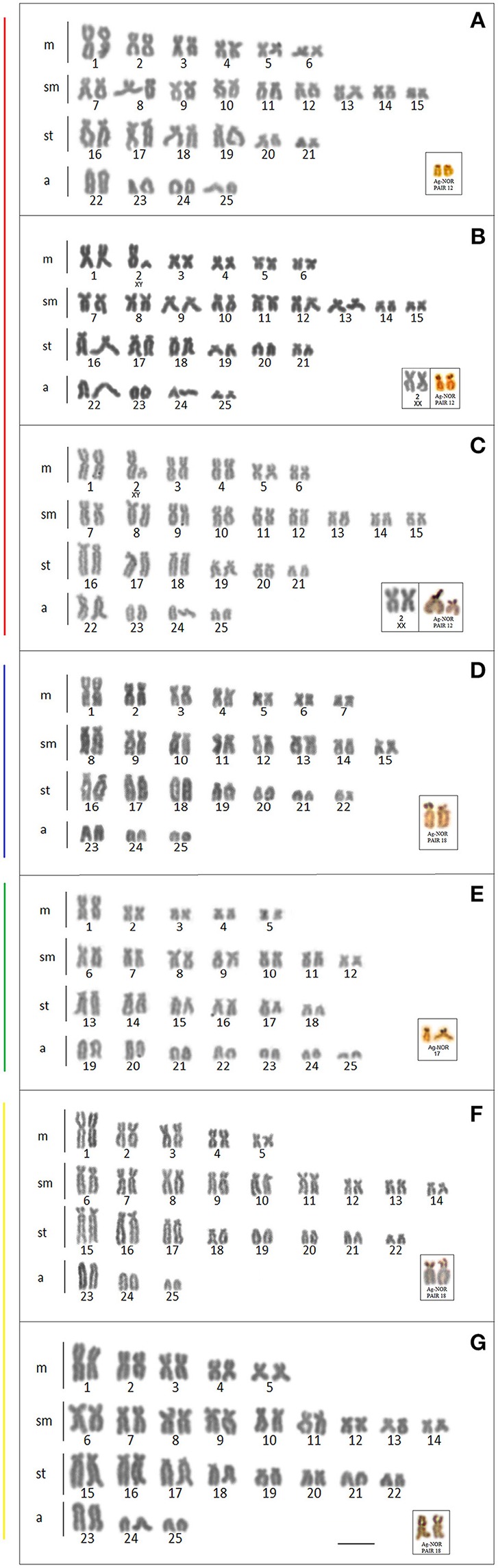
Karyotype of the *Ancistrus* populations sampled in different rivers of the Paraná basin, stained with Giemsa. The configuration of the silver nitrate-stained nucleolar organizing regions (Ag-NORs) are shown in the box. Each color of the side bars represents one of the evolutionary lineages recovered in the analysis, according to Figure 2. **(A)** L1: Mourão River; **(B)** L2: 19 Stream; **(C)** L3: Keller River; **(D)** L6: São Francisco Verdadeiro River; **(E)** L8: *Ancistrus cirrhosus*; **(F)** L9: Ocoí River; **(G)** L10: São Francisco Falso River. Bar = 10 μm.

**Figure 5:**
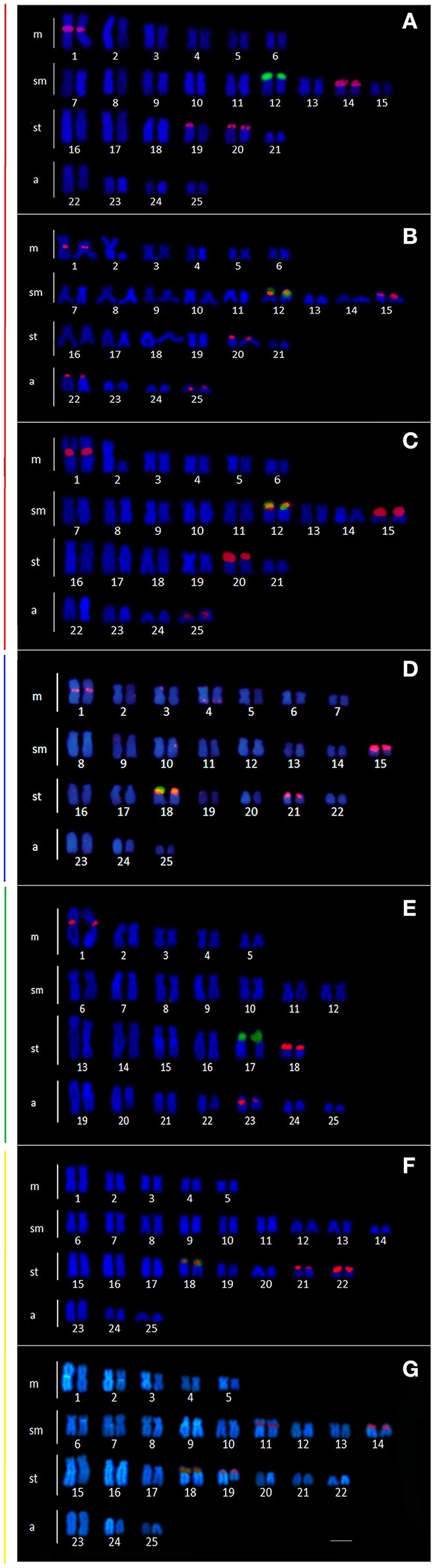
Chromosomes of the *Ancistrus* populations of species after dual color-FISH showing the 5S rDNA (red) and 18S rDNA (green) sites. Each color of the side bars represents one of the evolutionary lineages recovered in the analysis, according to Figure 2. **(A)** L1: Mourão River; **(B)** L2: 19 Stream; **(C)** L3: Keller River; **(D)** L6: São Francisco Verdadeiro River; **(E)** L8: *Ancistrus cirrhosus*; **(F)** L9: Ocoí River; **(G)** L10: São Francisco Falso River. Bar = 10 μm.

Cytogenetic data were obtained for the populations from the Mourão, Keller, São Francisco Verdadeiro, São Francisco Falso, and Ocoi Rivers, and 19 Stream (Brazil), and Arroyo San Juan (Argentina). All the specimens analyzed presented a diploid number of 2n = 50 chromosomes, although four distinct karyotype formulae were detected (Table 3, Figure 4), together with variation in the location of the 18S and 5S rDNA sites (Figure 5). Interestingly, these formulae corresponded strongly with the major clades recovered in our genetic analysis, with exception of the clade III that have no karyotype available. A chromosome heteromorphism found in all the males of Stream 19 (L2) and Keller River (L3) populations were consistent with an XX/XY system. The X chromosome is a large metacentric (second pair) while the Y chromosome is a small subtelocentric (Figure 4).

**Table 3:** Cytogenetic parameters of the *Ancistrus* populations sampled in the different rivers of the Paraná basin.

***Ancistrus* population**	**Clade**	**Karyotype formula**	**Ag-NOR**	**rDNA sites**	
				**18S**	**5S**
Mourão River (L1)	I	12m+18sm+12s+8a	12 (sm)	12 (sm)	1 (m), 14 (sm), 19 (st)^**^, 20 (st)
Stream 19 (L2)	I	12m+18sm+12s+8a-♀	12 (sm)	12 (sm)^*^	1 (m), 12 (sm)^*^, 15 (sm), 20 (st), 25(a)
		11m+18sm+13s+8a-♂		
Keller River (L3)	I	12m+18sm+12s+8a-♀	12 (sm)	12 (sm)^*^	1 (m), 12 (sm)^*^, 15 (sm), 20 (st), 22(a), 25(a)
		11m+18sm+13s+8a-♂		
São Francisco Verdadeiro River (L6)	II	14m+16sm+14st+6a	18 (st)	18 (st)^*^	1 (m), 15 (sm), 18 (st)^*^, 21 (st)
Arroyo San Juan (L8)	III	10m+14sm+12st+14a	17 (st)	17 (st)	1 (m), 18 (st), 23(a)
Ocoí River (L9)	IV	10m+18sm+16st+6a	18 (st)	18 (st)^*^	18 (st)^*^, 21 (st), 22 (st)
São Francisco Falso River (L10)	IV	10m+18sm+16st+6a	18 (st)	18 (st)^*^	11 (sm), 14 (sm)18 (st)^*^, 19 (st)

* Synteny between the 18S and 5S rDNA sites;

** *in only one homolog; m, metacentric; sm, submetacentric; st, subtelocentric; a, acrocentric; ♂, male; ♀, female*.

**Figure 6:**
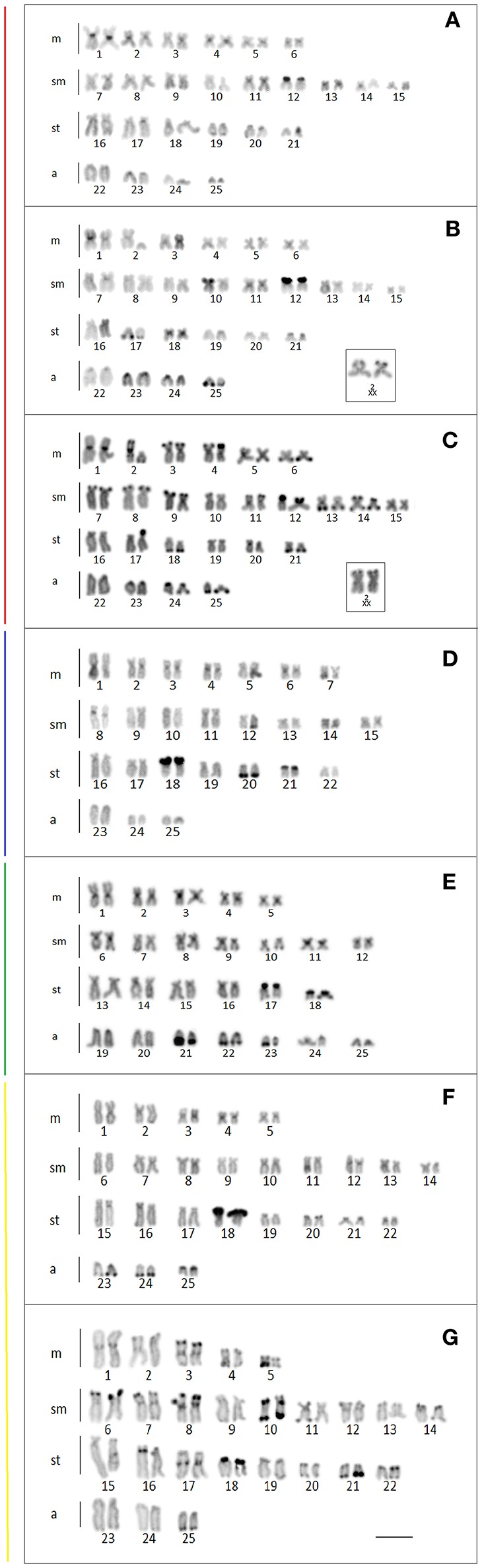
Karyotypes of the *Ancistrus* populations showing the distribution of the heterochromatin after C-banding. Each color of the side bars represents one of the evolutionary lineages recovered in the analysis, according to Figure 2. **(A)** L1, Mourão River; **(B)** L2, 19 Stream; **(C)** L3, Keller River; **(D)** L6, São Francisco Verdadeiro River; **(E)** L8, *Ancistrus cirrhosus*; **(F)** L9, Ocoí River; **(G)** L10, São Francisco Falso River. Bar = 10 μm.

The analysis of the heterochromatin revealed C-positive blocks at the pericentromeric and subterminal positions in a number of different chromosomal pairs of the populations allocated to clade I, with a conspicuous block coinciding with the NOR site of pair 12 in all karyotypes (Figures 6A–C). The X chromosome (pair 2, metacentric) from 19 Stream had a C-positive block in the pericentromeric region, while we detected heterochromatin blocks in the pericentromeric, interstitial, and subterminal regions of the X chromosome from Keller River. The Y chromosome of the Keller River population had a weak heterochromatin block in the subterminal region of the long arm, which was not found in the Y chromosome from 19 Stream (Figures 6B,C). In clades II, IV, and V, considerable variation was found in the amount and distribution of constitutive heterochromatin, which was concentrated primarily in the pericentromeric and subterminal regions of the chromosomes (Figures 6D–G). In the population from the São Francisco Falso River (clade V), in addition, we detected an interstitial heterochromatin block in pair 17, as well as a much larger amount of heterochromatin distributed throughout the chromosomes in comparison with the other populations (Figure 6G). Conspicuous blocks of heterochromatin were also found in the nucleolar pairs of all the populations analyzed in clades II and IV (pair 18) (Figures 6D,E).

The base-specific fluorochrome staining enhanced the distinctive composition of some heterochromatin blocks. In particular, staining with Chromomycin A3 revealed a richness of G and C in the subterminal and pericentromeric heterochromatin blocks of all the populations analyzed, and provides additional features for the comparative analysis. As expected, conspicuous C-positive areas were detected in the NOR-bearing chromosomes (pair 12, 17, and 18) (Figure S3).

## Discussion

### Molecular phylogenetic inferences reveal a number of distinct lineages in the paraná basin

The phylogenetic reconstructions and cytogenetic analyses presented in this study both detected the presence of five major clades among the *Ancistrus* populations surveyed, pointing to the existence of at least five lineages within the Paraná basin in Brazil previously undetected. Historically, all *Ancistrus* from the Paraná River basin were assigned to a single species, *A. cirrhosus*, which was described by Valenciennes (1836) from specimens collected in the Province of Misiones and Buenos Aires (Argentina), although the diagnostic traits are weakly defined (Langeani et al., 2007). Despite the morphological variations and wide geographic distribution, the *Ancistrus* populations of the Paraná basin have been identified invariably as *A. cirrhosus*. This fact has intrigued the ichthyologists with regard to the taxonomic status of many specimens, using only morphological and meristic characters. Therefore, the data obtained in the present study recovered at least five independent lineages (clades I–V) in Paraná basin. Given the proximity of the Arroyo San Juan to the type-locality of *A*. *cirrhosus*, this population (clade IV) was identified as nominal species *A*. *cirrhosus*. In this context, the other four clades (I, II, III, and V) can be categorized as “candidate species” in the terminology of Vieites et al. (2009). This interpretation is also supported by the results of the GMYC method, and the genetic distance detected among populations, considering a 2% threshold for interspecific differentiation.

The GMYC method has become one of the most popular tools for the delimitation of species based on single-locus data, and has been applied to the analysis of a number of poorly-known groups of organism (Barraclough et al., 2009; Monaghan et al., 2009; Marshall et al., 2011; Vuataz et al., 2011; Roxo et al., 2015). The GMYC method uses an ultrametric tree derived from the sequences to identify shifts in the branching rate from the Yule model (species) to the coalescent (population) process, with the algorithm computing the probability of splits between lineages in relation to speciation rates, thus identifying a cutoff value, at which species and populations split from one another (Powell, 2012). This approach was highly effective in the present study, allowing us to delimit five groups, which four corresponded exactly with the chromosomal data (unfortunately, no chromosomal data available to clade III), reinforcing the hypothesis that four “candidate species” exist in the Paraná River basin, besides *A*. *cirrhosus* as previously reported.

Armbruster (2004) found a sister group relationship between the Ancistrini and Pterygoplichthini tribes, which both belong to the subfamily Hypostominae. *Ancistrus* is the most species-rich genus of the tribe Ancistrini, although phylogenetic analyses of this genus are still scarce. Lujan et al. (2015) adopted a broad approach to the phylogenetic relationships of the Loricariidae, a Neotropical catfish family, but included few *Ancistrus* species, which reinforces the need for further research. Studies in systematics based on osteological (Schaefer, 1987) and molecular (Montoya-Burgos et al., 1998) data show that *Ancistrus* constitutes a monophyletic group of species. The lack of any robust phylogenetic tree for *Ancistrus* still limits our understanding of its intrageneric relationships, and more detailed analyses, with a more representative dataset, are needed to provide a more comprehensive understanding of the phylogenetic relationships of this genus.

### The chromosomal data reinforce the hypothesis of complete lineage divergence

Cytogenetic studies, together with molecular and biochemical analyses, may be useful for the identification of cryptic species (Nakayama et al., 2001; Milhomen et al., 2007). In the present study, in fact, the inclusion of a cytogenetic approach was crucial to the recognition of the “candidate species,” given that the distinct chromosomal formulae found in the four lineages (clades I, II, IV, and V) emphasizes their reciprocal monophyly and the lack of gene flow between them. The results obtained also complement the available cytogenetic data to the *Ancistrus* species, since these studies are currently restricted to the taxa found in the Paraguay (Mato Grosso) and Amazon (Manaus) basins (de Oliveira et al., 2007, 2008, 2009; Mariotto et al., 2011, 2013; Favarato et al., 2016; Prizon et al., 2016).

In the present study, a diploid number of 2n = 50 chromosomes was recorded in all the samples from the Paraná River basin. This number is within the range recorded for the genus, which vary from 2n = 34 in *Ancistrus cuiabae* to 2n = 54 in *Ancistrus claro* (Mariotto et al., 2009, 2013; Favarato et al., 2016; Prizon et al., 2016). Despite the homogeneity of the diploid number, the karyotype formula varied considerably among populations. The number of acrocentric chromosomes, for example, varied from three to four pairs in the populations of clades I, II, and V, to seven pairs in clade IV (Arroyo San Juan). This points evidences to the occurrence of chromosomal rearrangements, such as translocation and pericentric inversions, which did not affect the diploid number. Unfortunately, while we were able to identify these features, we were unable to trace the pathways of the transformations due to the lack of resolution in the internal topology of the *Ancistrus* lineages recognized here. In this case, further phylogenetic studies, based on a larger set of characters and a multi-locus dataset, may provide more definitive insights into the evolution of this group. Among all the ancistrinis species studied so far, the karyotypic evolution of *Ancistrus* is invariably associated with chromosomal rearrangements, which typically involve variation in the diploid number, given that this number ranges from 34 to 54 in this genus. As Artoni and Bertollo (2001) considered 2n = 54 to be the ancestral diploid number of the Loricariidae, karyotypic evolution in *Ancistrus* appears to have been associated with a reduction in the chromosome number. In fact, de Oliveira et al. (2009) suggested that the karyotypic evolution of this genus was predominantly involves by centric fusions.

In *Ancistrus* the occurrence of heteromorphic sex chromosomes has been well-documented in some species, including simple systems (Mariotto et al., 2004; Alves et al., 2006; Mariotto and Miyazawa, 2006) and multiples (de Oliveira et al., 2007, 2008) which also contributed to the karyotype evolution in the genus (Favarato et al., 2016). One peculiar feature observed in clade I was the heteromorphic sex chromosomes found in the populations of 19 Stream (L2) and the Keller River (L3), which are consistent with an XX/XY system. Surprisingly, however, this feature was not observed in the specimens from the Mourão River (L1), which were included in clade I and separated by low genetic distances from the populations of Stream 19 and the Keller River. The karyotypic formula of the Mourão River population differs from these two other populations only by the absence of the pair of sex chromosomes, and the GYMC analysis also identified these populations as a unique taxonomical unit. A similar result was obtained by Henning et al. (2011), combining cytogenetic and molecular data for different species of *Eigenmannia*, included two populations of *E. virescens* (Mogi-Guaçu and Tietê rivers), whose karyotypes with 2n = 38 differ by the presence of a pair sexual XX/XY (Tietê river population). The acrocentric X chromosome possesses a heterochromatinized distal region (Almeida-Toledo et al., 2001) and according to Henning et al. (2011), both populations (Mogi-Guaçu e Tietê rivers) were considered sister species. Furthermore, these authors concluded that seems likely that suppression of recombination in the homologous pair of acrocentric chromosomes and accumulation of heterochromatin on the X chromosome occurred after a recent geographical separation. In this context, we hypothesized that the heteromorphic sex chromosome found in the *Ancistrus* populations of 19 Stream and the Keller River represent a recent event which may have occurred after the geographical isolation of these populations from that of the Mourão River, together with the behavioral characteristics of these fish, which occupy specific microhabitats, form territories, and do not normally migrate (Power, 1984, 1990; Buck and Sazima, 1995). All these characteristics favor the fixation of chromosomal rearrangements and could be contribute to allopatric speciation on a micro scale (de Oliveira et al., 2009).

The hypothesis of the recent differentiation of the sex chromosome pair in the *Ancistrus* populations of clade I was also supported by the C-banding data. Heterochromatin is widely used in the identification of the sex chromosomes, and the addition or deletion of heterochromatin or the occurrence of a pericentric inversion involving one of the chromosomes have been postulated as important mechanisms in the origin of simple sexual chromosome systems in Neotropical fish (Almeida-Toledo et al., 2000). If we compare the Y chromosome of males from 19 Stream (L2) and Keller River (L3), it is possible to detect a discreet heterochromatin block in the subterminal region of the long arm of the Keller River males, which was not detected in the Y chromosomes from 19 Stream. The presence of the heteromorphic pair in the *Ancistrus* population of 19 Stream and Keller River suggests that chromosomal rearrangements (inversions), the loss of chromosomal material and, in the specific case of the Keller River, the presence of constitutive heterochromatin in the heteromorphic pair, may all be evidence of the recent origin of the Y chromosome, derived from a large metacentric, similar to the X chromosome. Pericentric inversions, followed by a loss of chromosomal material, have been suggest as a mechanism to explain the origin of the ZZ/ZW chromosome system in *Ancistrus* sp. Piagaçu (de Oliveira et al., 2007) and the XX/XY chromosome systems in *Ancistrus* sp. Purus and *Ancistrus* sp. Macoari (de Oliveira et al., 2009), given absence of the heterochromatin blocks in either the X or Y chromosomes.

The NOR mapping provided an excellent marker in the present study, being found in a single chromosome pair in each clade, a condition shared with most other *Ancistrus* species (Medeiros et al., 2016). The variation among clades in the NOR-bearing chromosome may be the result of chromosomal rearrangements occurring during chromosomal evolution. We also recorded synteny between the 18S and 5S rDNA sites in most populations of clades I, II, and V, except for the population from Mourão River (clade I). This synteny of the rDNA may represent the basal condition for the genus (Mariotto et al., 2011), as it is found in *A. claro* (2n = 54), although synteny of the ribosomal sites was not found in the population from Arroyo San Juan (clade IV). The position and distribution of the 5S rDNA sites varied considerably among the four clades, as they do in other *Ancistrus* species, occupying multiple sites in pericentromeric, interstitial, or terminal positions. This variation is considered to be an important reflection of the enormous karyotypic diversity found in the *Ancistrus*, which is seen as evidence of the apomorphic condition of this group (Medeiros et al., 2016). This variability, together with the disjunction of the ribosomal sites caused by rearrangements or mobile genetic elements appears to be a common condition among Neotropical fish species (de Oliveira et al., 2009). We observed heterochromatin in association with the ribosomal DNA sites (18 and 5S), recurrent characteristic in the karyotypes of Neotropical fishes (Vicari et al., 2003). The presence of heterochromatin, which contains large quantities of transposable elements (Dimitri et al., 2009) may facilitate transposition events, moving ribosomal genes around the genome (Moreira-Filho et al., 1984; Vicari et al., 2008; Gross et al., 2009, 2010). This may be one of the factors responsible for the presence of the multiple 5S ribosomal DNA sites found in the present study.

## Conclusion

The cytogenetic data available for the genus *Ancistrus* indicate a highly heterogeneous pattern of chromosome evolution, marked by Robertsonian and non-Robertsonian rearrangements. While we do not have an exact understanding of the mechanisms that determine these rearrangements in natural populations, their fixation may either initiate or contribute to the divergence process, with specific implications for the utility of chromosomal characters for phylogenetic inference (Sites and Kent, 1994). As in other fish groups, the sex chromosomes, present in some *Ancistrus* species, may have contributed to high rates of evolution. The inferences obtained in the present study from a combined approach of molecular and cytogenetic analyses further corroborate the taxonomic complexity of this genus. This approach was especially important due to the lack of diagnostic features in the morphology of these fishes. The hidden diversity of the study populations was nevertheless decoded successfully by the combined approach, which allowed us to differentiate five distinct lineages of *Ancistrus*, reinforcing the hypothesis of the presence of at least four candidate species in the upper Paraná River basin, besides of the *A. cirrhosus*, previously described. Finally, our findings reinforce the observation that the true diversity of the freshwater fish of the Neotropical has been underestimated and improve our understanding of a regional diversity within *Ancistrus* genus.

## Author contributions

AP: provided chromosomal and molecular data, and drafted the manuscript; DB: designed and coordinated the study of molecular data and helped draft the manuscript; HR and CZ: collected specimens from Paraná state and helped to identify the specimens; AF: collected and processed material of specimens from Arroyo San Juan, Argentina; LB-C, AC, and LB: assisted in the execution and analysis of chromosomal banding; AdBP-C: designed and coordinated the study of cytogenetic data and helped draft the manuscript. All authors have read and approved the final manuscript.

### Conflict of interest statement

The authors declare that the research was conducted in the absence of any commercial or financial relationships that could be construed as a potential conflict of interest.

## Supplementary material

Figure S1 Topology inferred from the Bayesian analysis performed with the software Mr. Bayes 3.2.6. posterior probabilities are shown at each node. Scale bar represents the number of substitutions per site. The terminal species with alphanumerical identifiers were obtained from GenBank (Supplementary File 1). Codes: L1, Mourão River; L2, 19 Stream; L3, Keller River; L4, Patos River; L5, São João River; L6, São Francisco Verdadeiro River; L7, Arroyo Iguaçu; L8, *Ancistrus cirrhosus*; L9, Ocoí River; L10, São Francisco Falso River.

Figure S2 Chromosomes of the *Ancistrus* populations of species after CMA3 staining. Each color of the side bars represents one of the evolutionary lineages recovered in the analysis, according to Figure 2. **(a)** L1, Mourão River; **(b)** L2, 19 Stream; **(c)** L3, Keller River; **(d)** L6, São Francisco Verdadeiro River; **(e)** L8, *Ancistrus cirrhosus*; **(f)** L9, Ocoí River; **(g)** L10, São Francisco Falso River. Bar = 10 μm.

Figure S3 NJ dendrograma of the *Ancistrus* specimens. Node values = bootstrap test (1,000 pseudo replicas) are shown next to the branches. The terminal species with alphanumerical identifiers were obtained from GenBank (Supplementary File 1). Codes: L1, Mourão River; L2, 19 Stream; L3, Keller River; L4, Patos River; L5, São João River; L6, São Francisco Verdadeiro River; L7, Arroyo Iguaçu; L8, *Ancistrus cirrhosus*; L9, Ocoí River; L10, São Francisco Falso River.
