# Supplementary figures and images for: Hidden Diversity in the Populations of the Armored Catfish Ancistrus Kner, 1854 (Loricariidae, Hypostominae) from the Paraná River Basin Revealed by Molecular and Cytogenetic Data
Source: Front Genet. 2017 Nov 24;8:185. doi: 10.3389/fgene.2017.00185 (PMC5705632; doi:10.3389/fgene.2017.00185)

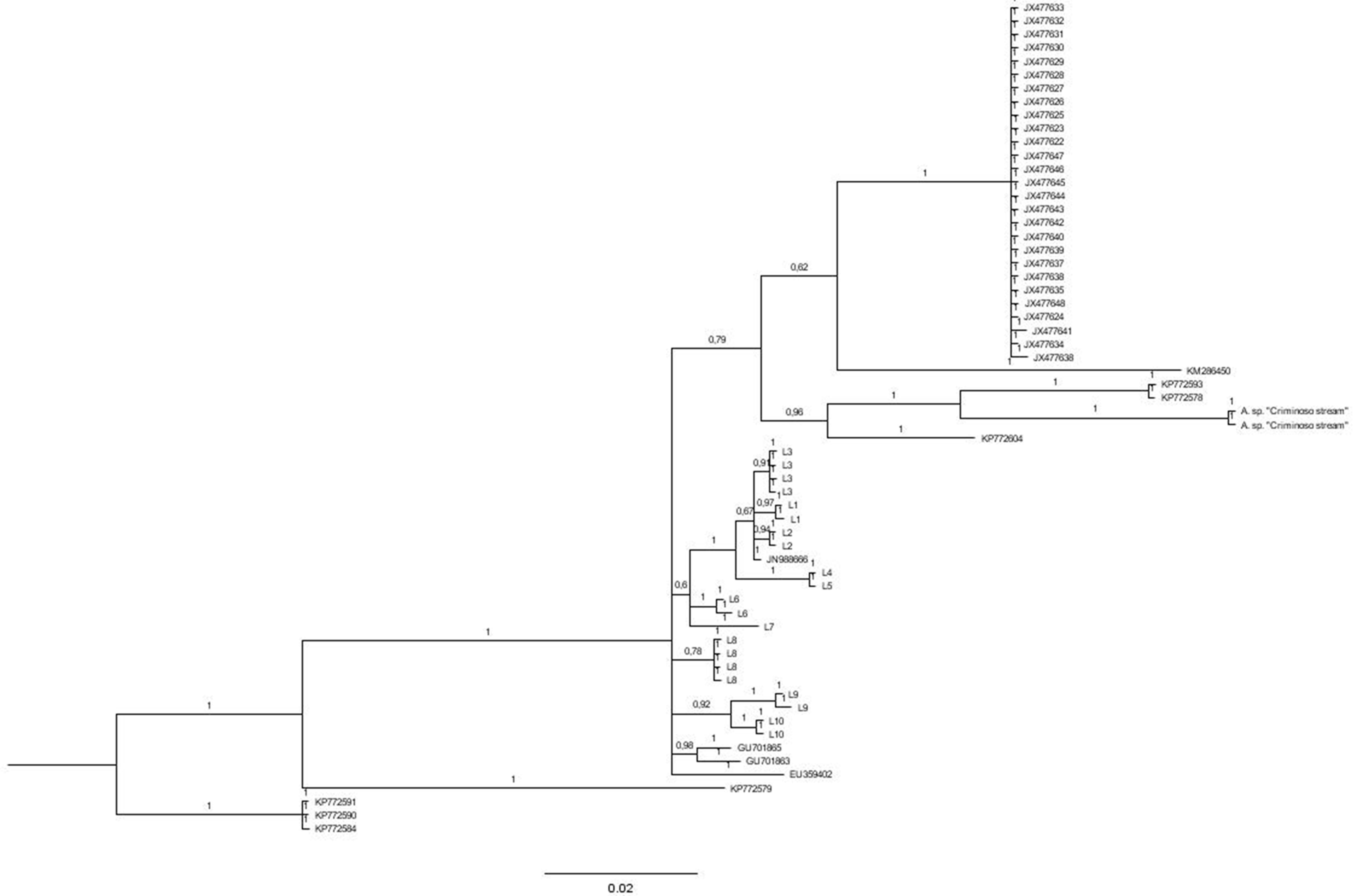

Supplement: Figure S1 — Topology inferred from the Bayesian analysis performed with the software Mr. Bayes 3.2.6. posterior probabilities are shown at each node. Scale bar represents the number of substitutions per site. The terminal species with alphanumerical identifiers were obtained from GenBank (Supplementary File 1). Codes: L1, Mourão River; L2, 19 Stream; L3, Keller River; L4, Patos River; L5, São João River; L6, São Francisco Verdadeiro River; L7, Arroyo Iguaçu; L8, Ancistrus cirrhosus; L9, Ocoí River; L10, São Francisco Falso River. [file Image1.TIF]

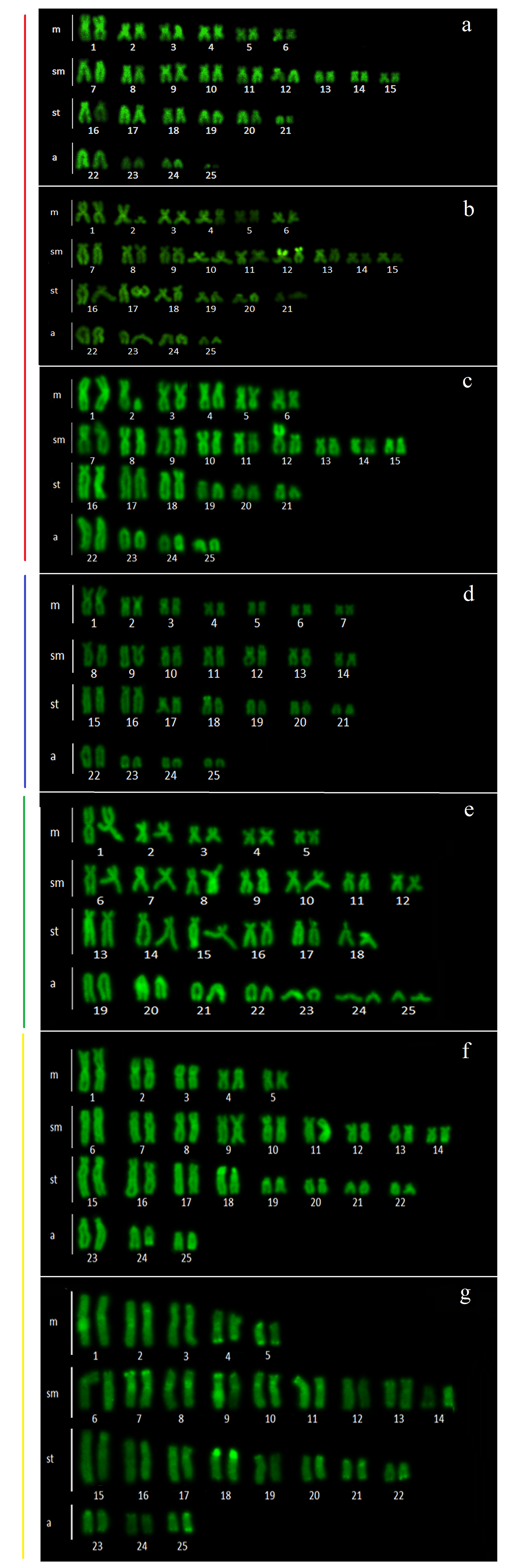

Supplement: Figure S2 — Chromosomes of the Ancistrus populations of species after CMA3 staining. Each color of the side bars represents one of the evolutionary lineages recovered in the analysis, according to Figure 2. (a) L1, Mourão River; (b) L2, 19 Stream; (c) L3, Keller River; (d) L6, São Francisco Verdadeiro River; (e) L8, Ancistrus cirrhosus; (f) L9, Ocoí River; (g) L10, São Francisco Falso River. Bar = 10 μm. [file Image2.TIF]
