# Supplementary material for: Hidden Diversity in the Populations of the Armored Catfish Ancistrus Kner, 1854 (Loricariidae, Hypostominae) from the Paraná River Basin Revealed by Molecular and Cytogenetic Data
Source: Front Genet. 2017 Nov 24;8:185. doi: 10.3389/fgene.2017.00185 (PMC5705632; doi:10.3389/fgene.2017.00185)
Supplement: Supplementary file 4 [file Table1.DOC]

**Supplementary file 1 (S1):** Details (species, voucher number, sample locality, accession number and authors) of the sequences obtained in

this work and from GenBank used for phylogenetic inferences.

| **Species** | **Voucher** | **Locality** | **Access** | **Reference** |
| --- | --- | --- | --- | --- |
| *Ancistrus cirrhosus*  *Ancistrus cirrhosus*  *Ancistrus cirrhosus*  *Ancistrus* sp*.*  [*Ancistrus brevipinnis*](http://www.ncbi.nlm.nih.gov/Taxonomy/Browser/wwwtax.cgi?id=510750)  *Ancistrus* sp.  *Ancistrus* sp.  *Ancistrus* sp.  *Ancistrus* sp.  *Ancistrus* sp*.*  *Ancistrus* sp.  *Ancistrus* sp.  *Ancistrus cryptophthalmus*  *Ancistrus cryptophthalmus*  [*Ancistrus cryptophthalmus*](http://www.ncbi.nlm.nih.gov/Taxonomy/Browser/wwwtax.cgi?id=1280074)  *Ancistrus cryptophthalmus*  *Ancistrus cryptophthalmus*  *Ancistrus cryptophthalmus*  *Ancistrus cryptophthalmus*  *Ancistrus cryptophthalmus*  *Ancistrus* sp.  *Ancistrus cryptophthalmus*  *Ancistrus cryptophthalmus*  *Ancistrus cryptophthalmus*  *Ancistrus cryptophthalmus*  *Ancistrus cryptophthalmus*  *Ancistrus cryptophthalmus*  *Ancistrus cryptophthalmus*  *Ancistrus cryptophthalmus*  *Ancistrus* sp*.*  *Ancistrus* sp*.*  *Ancistrus* sp*.*  *Ancistrus* sp  *Ancistrus dolichopterus*  *Ancistrus dolichopterus*  *Ancistrus* sp. “córrego Criminoso”  *Ancistrus* sp. “rio Mourão”  *Ancistrus* sp. “córrego 19”  *Ancistrus* sp. “rio Keller”  *Ancistrus* sp. “rios dos Patos”  *Ancistrus* sp. “rio São João”  *Ancistrus* sp. “rio SFVerdadeiro”  *Ancistrus* sp. “Arroyo Iguaçu”  *Ancistrus* *cirrhosus*  *Ancistrus* sp. “rio Ocoí”  *Ancistrus* sp. “rio SFFalso”  *Lasiancistrus schomburgkii*  *Pseudolithoxus* sp.  *Pseudolithoxus* sp.  *Pseudolithoxus* sp. | LBP-35987LBP-35959LBPV-34759Ex51H11 MCP 21246  INPA 43862  INPA 43877  INPA 43877  NUP 12018  NUP 11993  NUP 13646  NUP 18794  NUP 15537  NUP 15812  NUP 15146  NUP 15250  NUP 18795  NUP 4729  NUP 15145  INPA 43886  INPA 43889  INPA 43889  INPA 43889 | Brazil, Paraná, Ervalzinho/Marquinho  Brazil, Paraná, Nova Laranjeiras  Brazil, Paraná, Marabá  Germany  Brazil, Pará, Lower Nhamundá River  Brazil, Pará, Lower Nhamundá River  Brazil, Pará, Lower Nhamundá River  Brazil, Mato Grosso do Sul, Coxim  Brazil, Paraná, Campo Mourão  Brazil, Paraná, Paraíso do Norte  Brazil, Paraná, Marialva  Brazil, Paraná, Prudentópolis  Brazil, Paraná, Prudentópolis  Brazil, Paraná, Toledo  Brazil, Paraná, Marechal Cândido Rondon  Brazil, Misiones, Posadas  Brazil, Paraná, Medianeira  Brazil, Paraná, Vera Cruz do Oeste  Brazil, Pará, Lower Nhamundá River  Brazil, Pará, Lower Nhamundá River  Brazil, Pará, Lower Nhamundá River  Brazil, Pará, Lower Nhamundá River | GU701865  GU701863  JN988666  KM286450  EU359402  JX477648  JX477647  JX477646  JX477645  JX477644  JX477643  JX477642  JX477641  JX477640  JX477639  JX477638  JX477637  JX477636  JX477635  JX477634  JX477633  JX477632  JX477631  JX477630  JX477629  JX477628  JX477627  JX477626  JX477625  JX477624  JX477623  JX477622  KP772604  KP772593  KP772578  KP772579  KP772591  KP772590  KP772584 | Pereira et al. 2013  Pereira et al. 2013  Pereira et al., 2013  Knebelsberger et al. 2015  Cramer et al. 2007  Carvalho D.C. 2012  Carvalho D.C. 2012  Carvalho D.C. 2012  Carvalho D.C. 2012  Carvalho D.C. 2012  Carvalho D.C. 2012  Carvalho D.C. 2012  Carvalho D.C. 2012  Carvalho D.C. 2012  Carvalho D.C. 2012  Carvalho D.C. 2012  Carvalho D.C. 2012  Carvalho D.C. 2012  Carvalho D.C. 2012  Carvalho D.C. 2012  Carvalho D.C. 2012  Carvalho D.C. 2012  Carvalho D.C. 2012  Carvalho D.C. 2012  Carvalho D.C. 2012  Carvalho D.C. 2012  Carvalho D.C. 2012  Carvalho D.C. 2012  Carvalho D.C. 2012  Carvalho D.C. 2012  Carvalho D.C. 2012  Carvalho D.C. 2012  Collins R.A. et al. 2015  Collins R.A. et al. 2015  Collins R.A. et al. 2015  Prizon et al. 2016  Present study  Present study  Present study  Present study  Present study  Present study  Present study  Present study  Present study  Present study  Collins R.A. et al. 2015  Collins R.A. et al. 2015  Collins R.A. et al. 2015  Collins R.A. et al. 2015 |

**References:**

1. Pereira LHG, Maia GMG, Hanner R, Foresti F, Oliveira C. Can DNA barcoding accurately discriminate megadiverse Neotropical freshwater fish fauna? BMC Genetics. 2013, 14: 20.
2. Knebelsberger T, Dunz AR, Neumann D, Geiger MF. Molecular diversity of Germany's freshwater fishes and lampreys assessed by DNA barcoding. Mol Ecol Resour. 2015; 15 (3): 562-572.
3. Cramer CA, Liedke AMR, Bonatto SL, Reis RE. The phylogenetic relationships of the Hypoptopomatinae and Neoplecostominae (Siluriformes: Loricariidae) as inferred from mitochondrial cytochrome c oxidase I sequences. Bull Fish Biol. 2007; 9(1/2): 51-59.
4. Carvalho DC. *Ancistrus* molecular taxonomy. Unpublished. 2012.
5. Collins RA, Ribeiro ED, Machado VN, Farias IP, Hrbek T. A preliminary inventory of the catfishes of the lower Rio Nhamunda, Brazil (Ostariophysi, Siluriformes). Unpublished. 2015.
6. [Prizon AC](https://www.ncbi.nlm.nih.gov/pubmed/?term=Prizon AC%5BAuthor%5D&cauthor=true&cauthor_uid=28123683), [Borin-Carvalho LA](https://www.ncbi.nlm.nih.gov/pubmed/?term=Borin-Carvalho LA%5BAuthor%5D&cauthor=true&cauthor_uid=28123683), [Bruschi DP](https://www.ncbi.nlm.nih.gov/pubmed/?term=Bruschi DP%5BAuthor%5D&cauthor=true&cauthor_uid=28123683), [Ribeiro MO](https://www.ncbi.nlm.nih.gov/pubmed/?term=Ribeiro MO%5BAuthor%5D&cauthor=true&cauthor_uid=28123683), [Barbosa LM](https://www.ncbi.nlm.nih.gov/pubmed/?term=Barbosa LM%5BAuthor%5D&cauthor=true&cauthor_uid=28123683), [Ferreira GE](https://www.ncbi.nlm.nih.gov/pubmed/?term=Ferreira GE%5BAuthor%5D&cauthor=true&cauthor_uid=28123683), [Cius A](https://www.ncbi.nlm.nih.gov/pubmed/?term=Cius A%5BAuthor%5D&cauthor=true&cauthor_uid=28123683), [Zawadzki CH](https://www.ncbi.nlm.nih.gov/pubmed/?term=Zawadzki CH%5BAuthor%5D&cauthor=true&cauthor_uid=28123683), [Portela-Castro AL](https://www.ncbi.nlm.nih.gov/pubmed/?term=Portela-Castro AL%5BAuthor%5D&cauthor=true&cauthor_uid=28123683). Cytogenetic data on *Ancistrus* sp. (Siluriformes, Loricariidae) of the Paraguay River basin (MS) sheds light on intrageneric karyotype diversification. [Comp Cytogenet.](https://www.ncbi.nlm.nih.gov/pubmed/28123683) 2016; 10(4): 625-636.
